# An 11-Gene Signature Based on Treatment Responsiveness Predicts Radiation Therapy Survival Benefit Among Breast Cancer Patients

**DOI:** 10.3389/fonc.2021.816053

**Published:** 2022-01-06

**Authors:** Junjie Shen, Derui Yan, Lu Bai, Ruirui Geng, Xulun Zhao, Huijun Li, Yongfei Dong, Jianping Cao, Zaixiang Tang, Song-bai Liu

**Affiliations:** ^1^ Department of Biostatistics, School of Public Health, Medical College of Soochow University, Suzhou, China; ^2^ Suzhou Key Laboratory of Medical Biotechnology, Suzhou Vocational Health College, Suzhou, China; ^3^ Jiangsu Key Laboratory of Preventive and Translational Medicine for Geriatric Diseases, Medical College of Soochow University, Suzhou, China; ^4^ School of Radiation Medicine and Protection and Collaborative Innovation Center of Radiation Medicine of Jiangsu Higher Education Institutions, Soochow University, Suzhou, China

**Keywords:** breast cancer, radio-sensitivity, prognosis gene signature, WGCNA, LASSO

## Abstract

**Purpose:**

We developed a strategy of building prognosis gene signature based on clinical treatment responsiveness to predict radiotherapy survival benefit in breast cancer patients.

**Methods and Materials:**

Analyzed data came from the public database. PFS was used as an indicator of clinical treatment responsiveness. WGCNA was used to identify the most relevant modules to radiotherapy response. Based on the module genes, Cox regression model was used to build survival prognosis signature to distinguish the benefit group of radiotherapy. An external validation was also performed.

**Results:**

In the developed dataset, MEbrown module with 534 genes was identified by WGCNA, which was most correlated to the radiotherapy response of patients. A number of 11 hub genes were selected to build the survival prognosis signature. Patients that were divided into radio-sensitivity group and radio-resistant group based on the signature risk score had varied survival benefit. In developed dataset, the 3-, 5-, and 10-year AUC of the signature were 0.814 (CI95%: 0.742–0.905), 0.781 (CI95%: 0.682–0.880), and 0.762 (CI95%: 0.626–0.897), respectively. In validation dataset, the 3- and 5-year AUC of the signature were 0.706 (CI95%: 0.523–0.889) and 0.743 (CI95%: 0.595–0.891). The signature had higher predictive power than clinical factors alone and had more clinical prognosis efficiency. Functional enrichment analysis revealed that the identified genes were mainly enriched in immune-related processes. Further immune estimated analysis showed the difference in distribution of immune micro-environment between radio-sensitivity group and radio-resistant group.

**Conclusions:**

The 11-gene signature may reflect differences in tumor immune micro-environment that underlie the differential response to radiation therapy and could guide clinical-decision making related to radiation in breast cancer patients.

## Introduction

The World Health Organization (WHO, https://www.who.int/) has announced on February 3, 2021 that, breast cancer has now overtaken lung cancer as the world’s mostly commonly-diagnosed cancer, based on statistics released by the International Agency for Research on Cancer (IARC) in December 2020. A month later, a major new collaborative effort, the Global Breast Cancer Initiative, was introduced by the WHO, with the objective of reducing global breast cancer mortality and highlighted renewed commitment to improve survival. Cancer prognosis is a major concern in clinical decision making and an important public health issue.

More than half of cancer patients require radiotherapy as part of primary treatment for cancer care and radiotherapy is frequently used to treat the most common types, such as breast cancer, lung cancer and gastric cancer (1–3). Generally, breast cancer patients have a long postoperative survival time with common adjuvant setting like chemotherapy and radiotherapy. However, due to the so called molecular heterogeneity of tumor, there are still many patients who may not benefit from radiation therapy but suffer from radiation-induced toxicity (4), although they may share the same clinical and pathological features. In the era of precision medicine, exploration of tumor radio-sensitivity at the genome level has appealed to much attention. Personalized radiotherapy regimens based on cancer biology have become increasingly important (5, 6). Hence, recent clinical guidelines emphasize the importance of using multi-genetic tests to select patients who should receive adjuvant therapy (6).

Commonly, the radio-sensitivity of a tumor can be determined at the cellular level. For example, if a tumor entity shrinks or dies after radiation therapy, the tumor can be considered “responsive” to radiotherapy. Then, we can analyze the difference of gene profile characteristics between the sensitive and non-sensitive types. One of these examples is the radio-sensitivity index (RSI, high index = radio-resistance) (7). A 10-gene signature was identified and used to build a rank-based linear regression algorithm to predict an intrinsic radio-sensitivity and was validated on independent breast cancer dataset (8). Another example is the 31-gene signature based on micro-array data from NCI-60 cancer cells (9).

However, many experiments cannot be done on human beings. Experiments on animals would not guarantee that the same conclusion can be drawn for humans. In the real clinical data analysis, treatment response could reflect sensitivity to radiotherapy of tumor patients’. According to varied treatment responses of patients, some radiotherapy-associated genes and lncRNAs are identified using bio-informatics approach and are utilized to predict prognosis of patients in several cancers (10, 11). Nevertheless, a treatment response usually reflects a short-term therapeutic effect and is not enough to reflect clinical benefits, such as a survival advantage (12). For tumor patients with a long-time survival (e.g., breast cancer patients), a preferable indicator of clinical benefits is progression-free survival (PFS). PFS is defined as the time from randomization to the time of disease progression, which is established by a discrete clinical or radio-logical assessment and also depends on the growth rate of a cancer (13). As less affected by subsequent treatments, palliative care, and comorbidities, PFS is a better alternative endpoint for overall survival (OS) and can be evaluated prior to determining survival benefit (12).

In this study, we used PFS as an alternative indicator of radiotherapy response in breast cancer patients with radiotherapy. Weighted correlation network analysis (WGCNA) was used to screen the most relevant modules to radiotherapy response between response and non-response groups. Based on the module genes, we developed a survival prognosis signature of breast cancer patients to distinguish the benefit group of radiotherapy. For precision medicine, our work offered more evidence and clues for using radiotherapy response related genes as potential signature to identify radio-sensitive for cancer patients or as targets that promote personalize radiation.

## Materials and Methods

### Data Sources

We downloaded gene expression RNA-seq and phenotype data of GDC TCGA Breast Cancer (BRCA) cohort from the UCSC Xena website (https://gdc.xenahubs.net). The RNA-seq data (version: 07-18-2019, n = 1,217) are standardized by Fragments Per Kilobase per Million (FPKM) and the unit is normalized as log2 (fpkm + 1). The mRNA and lncRNA expression data were extracted according to the GENCODE annotations database V38 (https://www.gencodegenes.org/). Phenotype data include clinical phenotype (version: 08-07-2019, n = 1,284) and survival data (version: 07-18-2019, n = 1,260).

The gene expression dataset was collated to exclude normal tissues and samples of metastatic tumors. After matching clinical information, we excluded samples of male breast cancer and unidentified gender. Next, we removed subjects with missing survival data or radiotherapy information. Patients with follow-up survival time less than 30 days were also eliminated. Finally, 937 patients were included in the study population. We adopted multivariable stepwise Cox regression analysis based on the AIC to identify major clinical influence factors for OS (See **Table 1**). Age, radiotherapy, chemotherapy, age, progesterone receptor (PR) status, N stage, and pathological stage were the impact factors of OS, which were reasonable.

**Table 1:** Associations of clinical variables with OS in BRCA (total N = 937).

		N	%	HR (95%CI)	*P*
Radiotherapy^*^	yes	534	57		
	no	403	43	2.348 (1.553, 3.551)	<0.001
Chemotherapy^*^	yes	761	90	1.000	
	no	86	10	1.987 (1.200, 3.290)	0.008
	unknown	90			
Age^*^	<60	503	54	1.000	
	≥60	434	46	2.139 (1.442, 3.173)	<0.001
Histology	IDC	663	71	1.000	
	ILC	187	20	0.872 (0.522, 1.458)	0.774
	others	86	9	1.592 (0.803, 3.152)	0.138
	unknown	1			
ER status	positive	699	78	1.000	
	negative	199	22	0.952 (0.509, 1.178)	0.877
	unknown	39			
PR status^*^	positive	607	68	1.000	
	negative	289	32	1.765 (1.178, 2.644)	0.006
	unknown	41			
T Stage	T1/T2	784	84	1.000	
	T3/T4	150	16	1.021 (0.585, 1.782)	0.942
	unknown	3			
N Stage^*^	N0/N1/N2	750	82	1.000	
	N3	169	18	1.597 (0.859, 2.970)	0.139
	unknown	18			
M Stage	M0	778	98	–	
	M1	18	2.0		
	unknown	141			
Pathological stage^*^	I/II	688	75	1.000	
	III/IV	230	25	3.129 (1.771, 5.530)	<0.001

IDC, infiltrating ductal carcinoma; ILC, infiltrating lobular carcinoma; ER, estrogen receptor; PR, progesterone receptor; TNM, tumor-node-metastasis stage.

^*^Clinical variables that were left after fast backward multivariate COX regression.

In addition, external validation was performed using E-TABM-158 dataset (n = 130) downloaded from the *ArrayExpress* database (https://www.ebi.ac.uk/arrayexpress/). This dataset includes information of transcription profiling of human breast cancer samples and clinical outcome (14). The inclusion and exclusion criteria of samples were the same as above.

### Study Design

**Figure 1:**
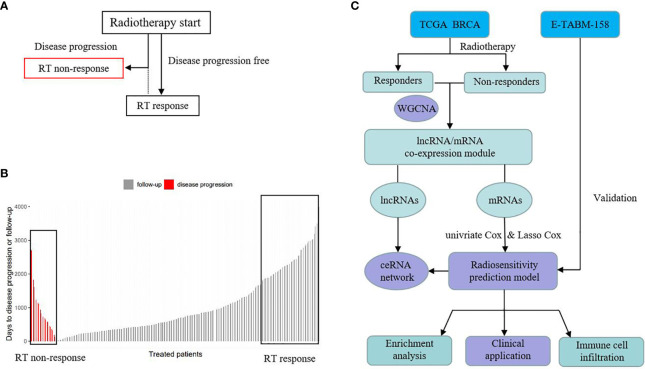
Schematic of study design. **(A)** The definition of RT response group and RT non-response group. **(B)** The rank of RT response group and RT non-response group based on time. **(C)** Analysis process.

**Figure 1** is the flow chart. In this study, we used TCGA BRCA as developed dataset. Patients with PFS happened after the start of radiotherapy (RT) and were defined as “RT non-response” group and those without a PFS event were defined as “RT response” group (See **Figure 1A**). In the 534 RT patients, there were 44 patients in the “RT non-response” group and 418 patients in the “RT response” group. Patients with missing RT time data were excluded. We ranked the patients based on their days to disease progression or follow-up time (**Figure 1B** **)**. Then, patients with the longer follow-up survival time in the “RT response” group were selected as control group to match main clinical factors (age, chemotherapy, PR status, pathological stage, and N Stage, see **Table S1**) with the “RT non-response” group using propensity score matching method.

After removing samples with outlier gene expression using hierarchical clustering analysis, 41 pairs of RT non-response/response samples were left. WGCNA was used to identify the most relevant mRNA and lncRNA modules to RT response between the two groups. Based on the relevant mRNA module genes, univariate Cox regression analysis was performed to screen survival related genes. Then common genes in both TACGA BRCA and E-TABM-158 were selected to build a survival prognosis signature model for breast cancer patients using Least Absolute Shrinkage and Selection Operator (LASSO) Cox regression model. This gene signature was evaluated in the developed dataset and validated in an external dataset. In addition, we also explored the related biological mechanisms (See **Figure 1C**).

### WGCNA Correlation Analysis

Weighted gene co-expression network analysis is a systems biology method to describe the correlation patterns among genes and clinical traits (15), which can be conducted using the *WGCNA* package in R (4.0.5) software. It is generally believed that genes with low amplitude variation and low expression do not play an important biological role in regulating body function and improving computational efficiency of WGCNA. Median absolute deviation (MAD) is a robust statistic used to describe the dissociation between samples (16). In this study, the top 5,000 mRNAs and lncRNAs with highest MAD were selected to perform WGCNA.

The WGCNA process is as follows. First, the value of the “soft” threshold parameter (beta) is estimated using the “pickSoftThreshold” function. The R-squared criterion of scale-free topology is set to 0.9. Then, Pearson correlation coefficients between genes are calculated using the expression data and the correlation matrix is converted to a weighted adjacency matrix based on beta. Next, a topological overlap matrix (TOM) is generated to describe the connection degree between genes. Genes with high connection degree are then grouped into the same module. The merge cut-off threshold is set to 0.2, which means that modules with a similarity higher than 0.8 are merged into one module (17).

After the relevant modules are grouped, principal component analysis (PCA) of the modules is performed. The first principal component (namely, eigengene) is extracted to represent the gene expression level within the module and is used for Pearson correlation analysis with clinical traits like RT response. Module with the strongest correlation to RT response and P-value <0.05 is considered associated with radio-sensitivity.

### Establishment of Prognostic Gene Signature

As mentioned above, univariate Cox regression model and LASSO Cox regression model with penalty parameter tuning conducted by 10-fold cross-validation were applied to build a radio-sensitivity related survival gene signature based on the relevant mRNA module genes. The risk score formula is as below:


(1) $$\text{Risk score}=\sum_{i=1}^{n}\text{Coef}(i)X(i)$$

where n is the number of genes in the prognostic prediction model, Coef(i) represents the coefficient, and X(i) means the relative genes expression level.

Risk score could be calculated using related gene expression value. The optimal cutoff value of risk score was determined using R package *survminer*. Patients were divided into low risk score group (radio-sensitive, RS) and high risk score group (radio-resistant, RR) based on the cutoff value of risk score. R packet *survival* was used to perform survival analysis between these two groups. Receiver operating characteristic (ROC) curves and its area under the curve (AUC) values were utilized to evaluate the specificity and sensitivity of the signature in a time-dependent manner using package *timeROC*. Calibration curves were used to evaluate the reliability and accuracy of the ROC curves. Lastly, an external validation of the survival gene signature was conducted using the above methods.

### Clinical Application

In order to evaluate the clinical application value of the survival prognosis gene signature, the gene signature was applied with relevant clinical characteristics to a stepwise multivariate Cox proportional hazards model. Multivariate ROC curves for gene signature and clinical factors were plotted. Then, a prognostic nomogram predicting 3-, 5-, and 10-year survival probability for BRCA patients in the RT group was constructed based on the Cox model. Further, clinical decision curve analysis (DCA) was performed based on several models to evaluate benefit value of survival prognosis gene signature. We tested the discrimination of the Cox model by Harrell’s concordance index (C-index) analysis.

### Functional Enrichment Analysis

We performed Gene ontology (GO) enrichment analysis of target module genes implemented using the R package *clusterprofiler*. GO enrichment analysis includes three ontologies, namely, biological process (BP), molecular function (MF), and cellular component (CC). The adjusted P-value <0.05 of GO enrichment analysis using the Benjamini–Hochberg method was considered statistically significant. The R package *GOplot* was used to visualize the GO enrichment data. Furthermore, the Database for Annotation, Visualization and Integrated Discovery (DAVID) online tool (https://david.ncifcrf.gov/tools.jsp) was used to collect more detailed biological function annotation information.

### CeRNA Network

Competing Endogenous RNAs (ceRNA) hypothesis reveals a novel mechanism of RNAs interaction. MiRNAs are known to cause gene silencing by binding mRNAs, while lncRNAs as ceRNAs can regulate gene expression by competitively binding miRNAs (18). We searched miRNAs binding with survival prognosis signature genes using two RNA interaction databases, namely, miRDB (http://mirdb.org/) and mirTarbase (http://mirtarbase.cuhk.edu.cn/), and the sum aggregate of these two databases was considered as the target miRNA to signature genes. Then the matched miRNAs were used to predicted interaction with their targeted lncRNA using another two RNA interaction databases starBase (https://starbase.sysu.edu.cn/) and lncBase (http://carolina.imis.athena-innovation.gr/diana_tools/web/index.php). The matched lncRNAs that were common in radio-sensitivity relevant lncRNA module were included into ceRNA construction. The R package *ggalluvial* was used for the visualization of the ceRNA network.

### Immune Cell Infiltration Analysis

Tumor-infiltrating immune cells are vital for cancer treatment and patient prognosis. To compare the difference of immune micro-environment between the radio-sensitive group and radio-resistant group, abundance tumor-infiltrating immune cells (TIICs) data was estimated and downloaded from the TIMER2.0 database (19) (http://timer.cistrome.org/). TIMER2.0 provides more robust estimation of immune infiltration levels for TCGA tumor profiles using state-of-the-art algorithms. The distributions of immune cells, including CD8^+^ T cells, CD4^+^ T cells, B cells, neutrophils, macrophages, and dendritic cells (DCs) were exhibited by a box-plot to explore the relationship between gene expression and immune infiltration from the two groups. In addition, we explored several immune checkpoint genes expression level between the two groups and compared the immune score using ESTIMATE method (20).

### Statistic Methods

In this study, all gene data were standardized into “Z-score” using function “covariates” in R packet *BhGLM*. R packet *glmnet* was used to perform LASSO regression model. Kaplan–Meier (K–M) curve was used to show the survival curves. Log-rank test evaluated the statistically significant differences of survival. Nomogram was plotted by using R package *rms*. Wilcoxon test was used to compare two groups with continuous variables that were non-normal. For missing clinical variable data, R packet *mice* (multiple imputation by chained equations) was used for multiple interpolation (21). All statistical analyses were performed using the R (4.0.5) software. A P-value of 0.05 was considered significant. All statistical tests were two-sided.

## Results

### WGCNA Correlation Analysis

**Figure 2:**
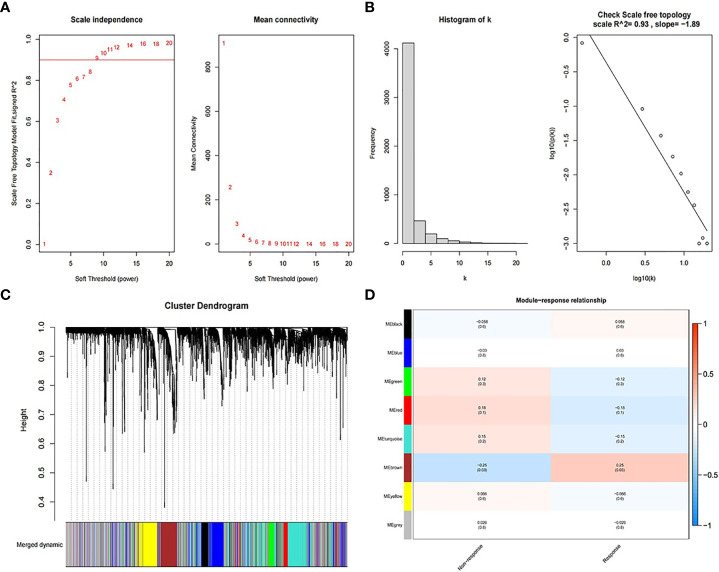
Process of searching the most relevant mRNA module to RT response. **(A)** Soft threshold for the construction of the co-expression network. **(B)** R-squared of scale-free topology. **(C)** Cluster dendrogram of merged dynamic modules. **(D)** The correlation coefficients between modules and RT response.

**Figure 2** shows the process of searching the most relevant mRNA module to RT response between 41 pairs of RT non-response/response samples using the top 5,000 mRNAs with highest MAD. The beta value for the construction of the co-expression network was set to 10 (**Figure 2A**). The obtained R-squared of scale-free topology was 0.93 (**Figure 2B**). After dynamic branch cut and modules merge process, WGCNA identified eight modules (**Figure 2C**) and calculated the coefficients associated with RT response (**Figure 2D**). MEbrown module with 534 genes, was most correlated to the RT response of patients. Similarly, the lncRNA modules relevant to RT response are shown in **Figure S1**.

### Establishment of Prognostic Gene Signature

**Figure 3:**
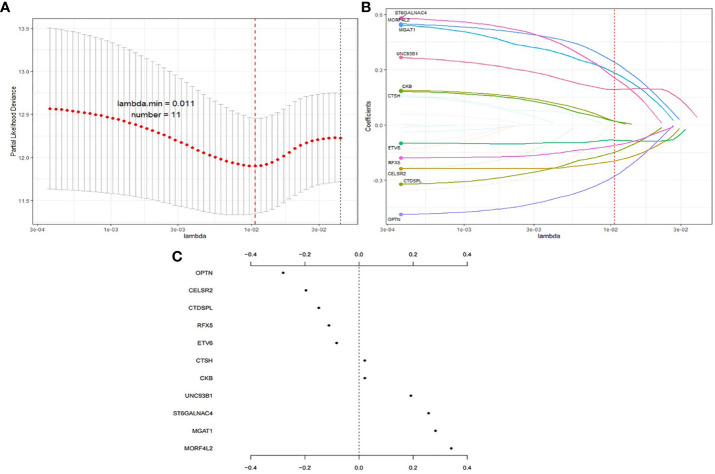
Process of gene signature construction using LASSO. **(A)** Penalty parameter tuning conducted by 10-fold cross-validation. **(B)** 11 hub genes with **(C)** their coefficients.

Among the 534 genes in MEbrown module, univariate Cox regression analysis screened 32 survival related genes and 22 genes were common in E-TABM-158 dataset. Then these 22 genes were thrown into the LASSO Cox regression model to build a survival prognosis signature in 534 RT BRCA patients. **Figure 3** shows the process of gene signature construction. With penalty parameter tuning conducted by 10-fold cross-validation, lambda parameter was set to 0.011 when partial likelihood deviance reached the minimum value (**Figure 3A**). According to the lambda, a number of 11 hub genes were selected (**Figure 3B**) with their coefficients (**Figure 3C**). Based on the formula (1), the risk score was calculated as follows:


$$\begin{array}{c} \text{Risk score}=0.022\times \text{CKB}+0.283\times \text{MGAT}1-0.149\times \text{CTDSPL}\\ +0.341\times \text{MORF}4\text{L}2-0.280\times \text{OPTN}+0.021\times \text{CTSH}\\ -0.111\times \text{CKB}-0.196\times \text{CELSR}2-0.083\times \text{ETV}6\\ +0.257\times \text{ST}6\text{GALNAC}4+0.192\times \text{UNC}93\text{B}1 \end{array}$$

**Figure 4:**
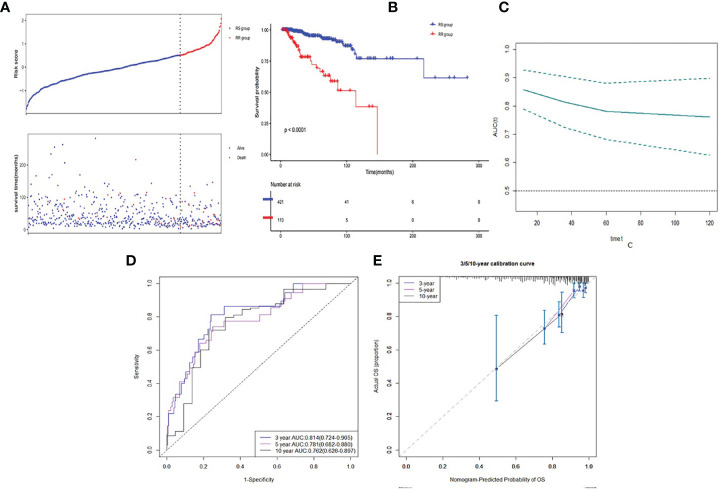
Discrimination ability of gene signature in developed dataset. **(A)** Low risk score group and high risk score group based on the cutoff value of risk score. **(B)** K–M curve of comparison for the RS and RR groups. **(C)** Time-dependent AUC of the risk score. **(D)** The 3-, 5- and 10-year AUC of the risk score. **(E)** Calibration plot for 3-, 5- and 10-year AUC of the risk score.

The optimal cutoff value of risk score was 0.515. Then patients were divided into low risk score group (RS group, n = 421) and high risk score group (RR group, n = 113) based on the cutoff value of risk score (See **Figure 4A**). RS group had a much higher survival rate (P <0.001) compared to RR group (**Figure 4B**). The median survival time of RS group was 1,043 days (Q1:588, Q3:2,041) whereas the RR group was 760 days (Q1:504, Q3:1,308). Time-dependent AUC curve showed that our survival prognosis signature worked well and robust (**Figure 4C**). The 3-, 5-, and 10-year AUC of the risk score were 0.814 (CI95%: 0.742–0.905), 0.781 (CI95%: 0.682–0.880), and 0.762 (CI95%: 0.626–0.897), respectively (**Figure 4D**). Calibration plot showed a good reliability and accuracy of the ROC curve (**Figure 4E**).

**Figure 5:**
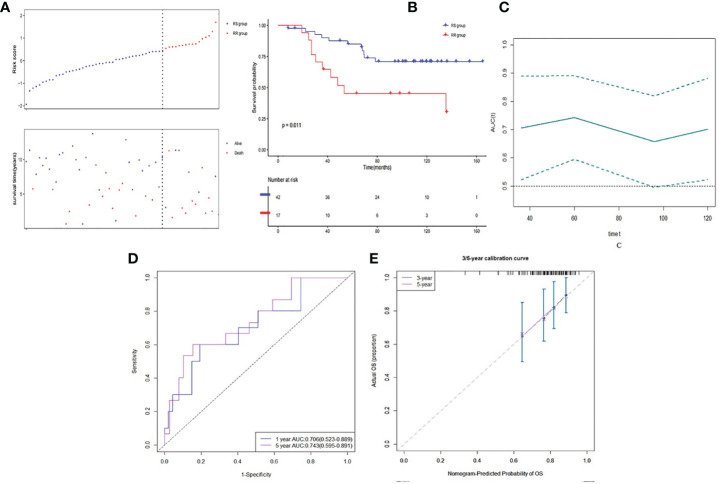
Discrimination ability of gene signature in validation dataset. **(A)** Low risk score group and high risk score group based on the cutoff value of risk score. **(B)** K–M curve of comparison for the RS and RR groups. **(C)** Time-dependent AUC of the risk score. **(D)** The 3- and 5-year AUC of the risk score. **(E)** Calibration plot for 3- and 5-year AUC of the risk score.

In the validation dataset, E-TABM-158, risk score was calculated based on the same method in the RT patients (n = 59). Patients were also divided into the RS group (n = 42) and the RR group (n = 17) (See **Figure 5A**). Similarly, RS group had a statistically significant higher survival rate (P = 0.011) compared to RR group (**Figure 5B**). Time-dependent AUC curve fluctuated around 0.7 (**Figure 5C**). The 3- and 5-year AUC of the risk score were 0.706 (CI95%: 0.523–0.889) and 0.743 (CI95%: 0.595–0.891) (**Figure 5D**). Calibration plot seemed good as well (**Figure 5E**). Due to limited observed samples, we did not predict the 10-year survival in this dataset.

**Figure 6:**
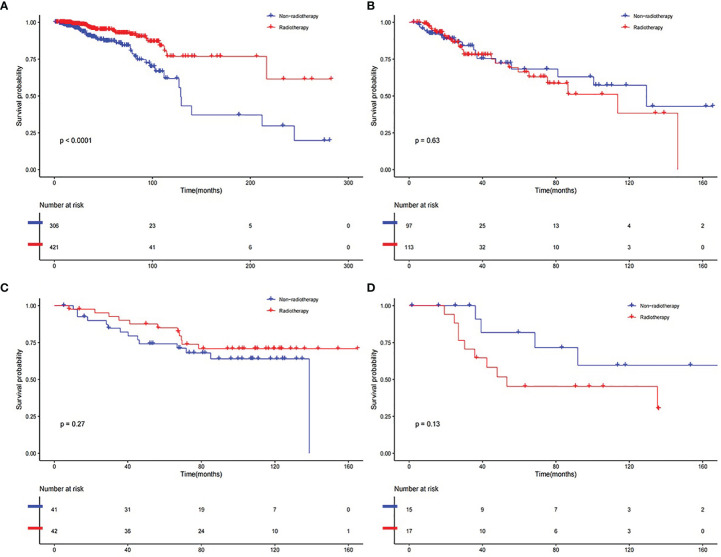
K–M curve of comparison for the RT and Non-RT groups. **(A)** RS group in developed dataset. **(B)** RR group in developed dataset. **(C)** RS group in validation dataset. **(D)** RR group in validation dataset.

Lastly, we calculated the risk score of the patients in the whole samples in both developed and validation datasets. In TCGA BRCA dataset, the RS group received RT (n = 421) had a much higher survival rate (P <0.001) compared to the RS group without RT (n = 306) (**Figure 6A**). The RR group received RT (n = 113) had no better survival rate (P = 0.63) compared to the RR group without RT (n = 97) (**Figure 6B**). The RS patients gained additional survival benefit after receiving RT. This phenomenon was not observed in the validation group, though the RS group that received RT (n = 42) seemed to have a slightly higher survival (P = 0.27) compared to the RS group without RT (n = 41) (**Figure 6C**). While the RR group that received RT (n = 17) was prone to having lower survival (P = 0.13) compared to the RR group without RT (n = 15) (**Figure 6D**).

### Clinical Application

**Figure 7:**
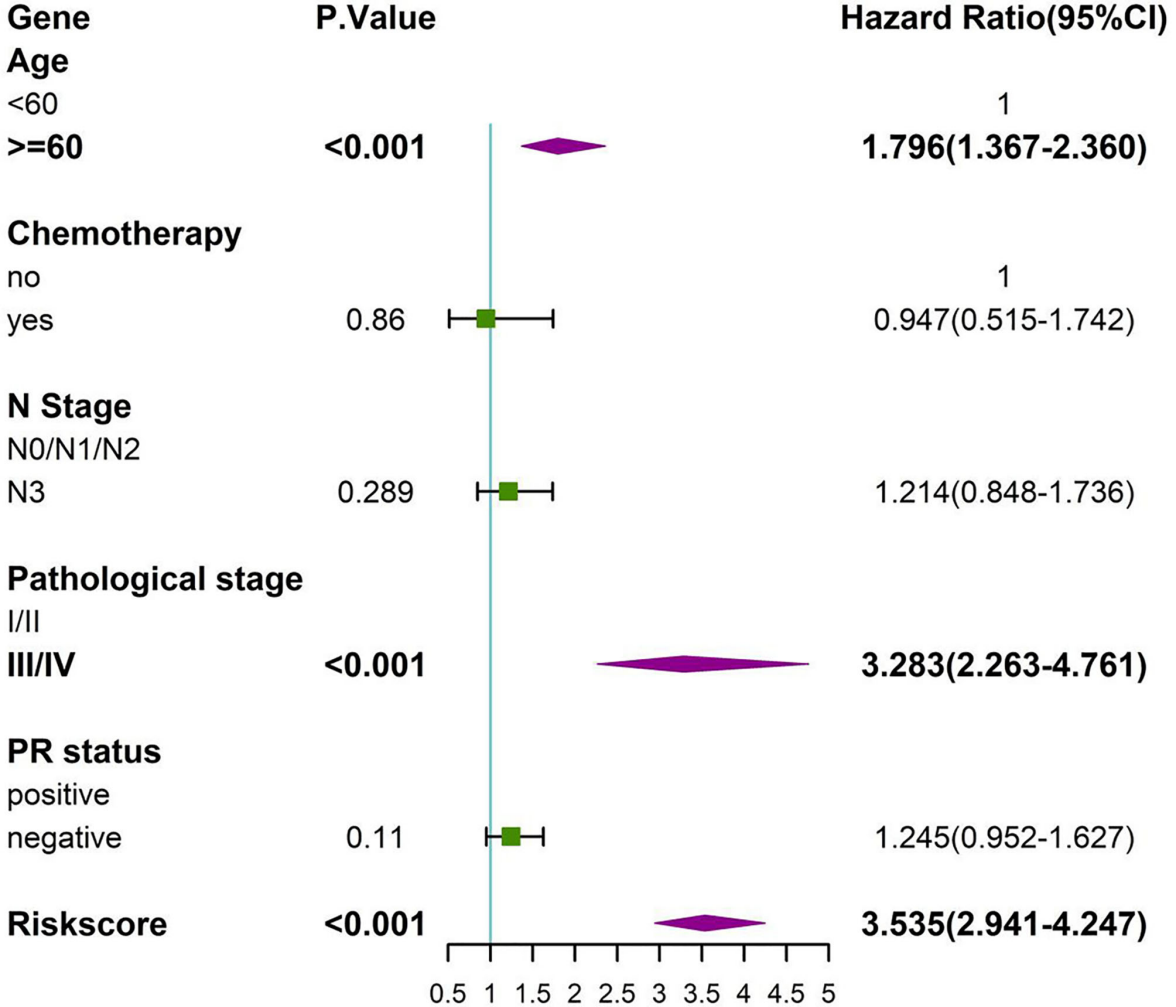
Forest plot for multivariate Cox model in RT BRCA.

**Figure 8:**
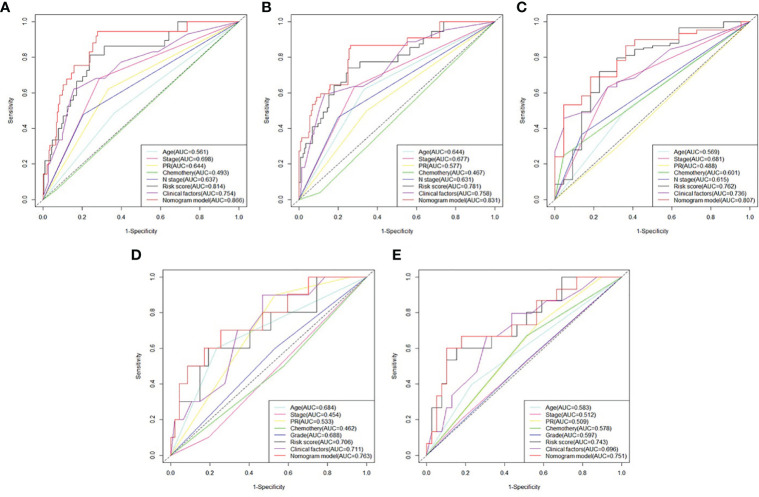
Comparison between survival prognosis gene signature and clinical factors. **(A)** Multivariate ROC curves for 3-, **(B)** 5-, and **(C)** 10-year survival in developed dataset. **(D)** Multivariate ROC curves for 3- and **(E)** 5-year survival in validation dataset.

The prognosis gene signature was applied with relevant clinical factors to a stepwise multivariate Cox proportional hazards model in RT TCGA BRCA patients. **Figure 7** shows that the risk score was an independent factor to OS. Each unit increased in the risk score, was associated with a 3.535-fold (CI95%: 2.941–4.247) increase in the risk of death. Multivariate ROC curves for 3-, 5-, and 10-year survival show that gene signature had better predictive power than relevant clinical factors (**Figures 8A–C**). The combined model of gene signature and clinical factors could reach over 0.8 of accuracy. Same conclusion was found in validation dataset (**Figures 8D, E**). The combined model could reach over 0.75 of accuracy.

**Figure 9:**
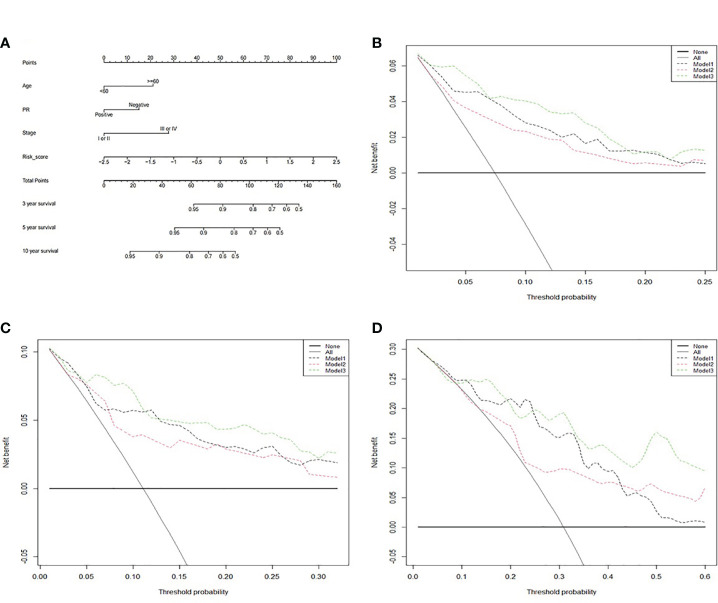
The clinical application value of the survival prognosis gene signature. **(A)** Nomogram for predicting 3-, 5-, and 10-year survival of RT BRCA patients. **(B)** DCA curve in 3-, **(C)** 5-, and **(D)** 10-year using three models.

Then, a prognostic nomogram predicting 3-, 5-, and 10-year survival rate for RT BRCA patients was constructed based on the Cox model using age, PR status, pathological stage and risk score (**Figure 9A**). With 1,000 cross-validation, the C-index of the nomogram based on the Cox model is 0.836. Further, clinical DCA was plotted based on several models at 3-, 5-, and 10-year (**Figures 9B–D**). Model1 was Cox model using risk score. Model2 was Cox model using clinical factors and Model3 was a mixed of clinical factors and risk score. Model3 had the maximum clinical net benefit. Model2 was better than Model1.

### Functional Enrichment Analysis

**Figure 10:**
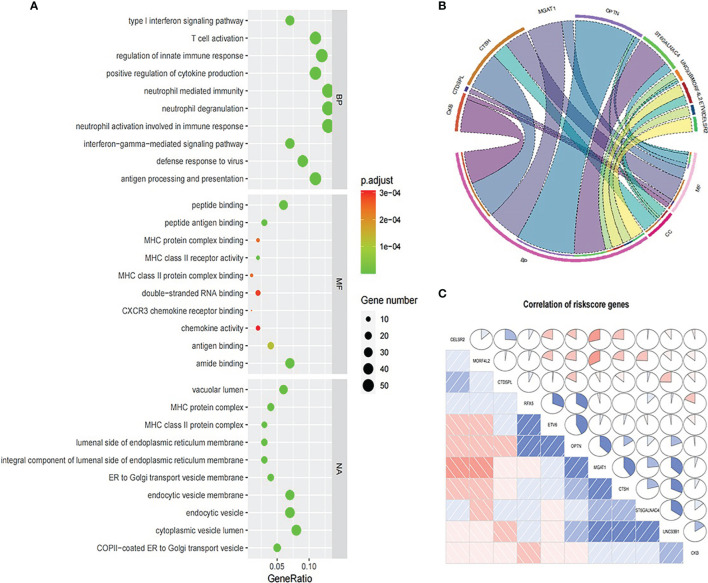
Functional enrichment analysis. **(A)** GO analysis for 534 genes in MEbrown module. **(B)** GO analysis for 11 genes in the prognosis signature. **(C)** Expression correlation between 11 prognosis signature genes.

We explored the functional enrichment of 534 genes in MEbrown module (See **Figure 10**). The module genes were mainly enriched in “T cell activation”, “regulation of innate immune response”, “neutrophil mediated immunity”, etc., immune-related processes in BP (**Figure 10A**). We also explored the functional enrichment of the 11 genes in the prognosis signature (**Figure 10B**). Most of these genes were separately enriched and mainly involved in BP. CTSH and UNC93B1 were enriched in the same ontology “adaptive immune response”. Correlation coefficient plot based on gene expression shows that the correlations between these genes were low (**Figure 10C**).

### CeRNA Network

**Figure 11:**
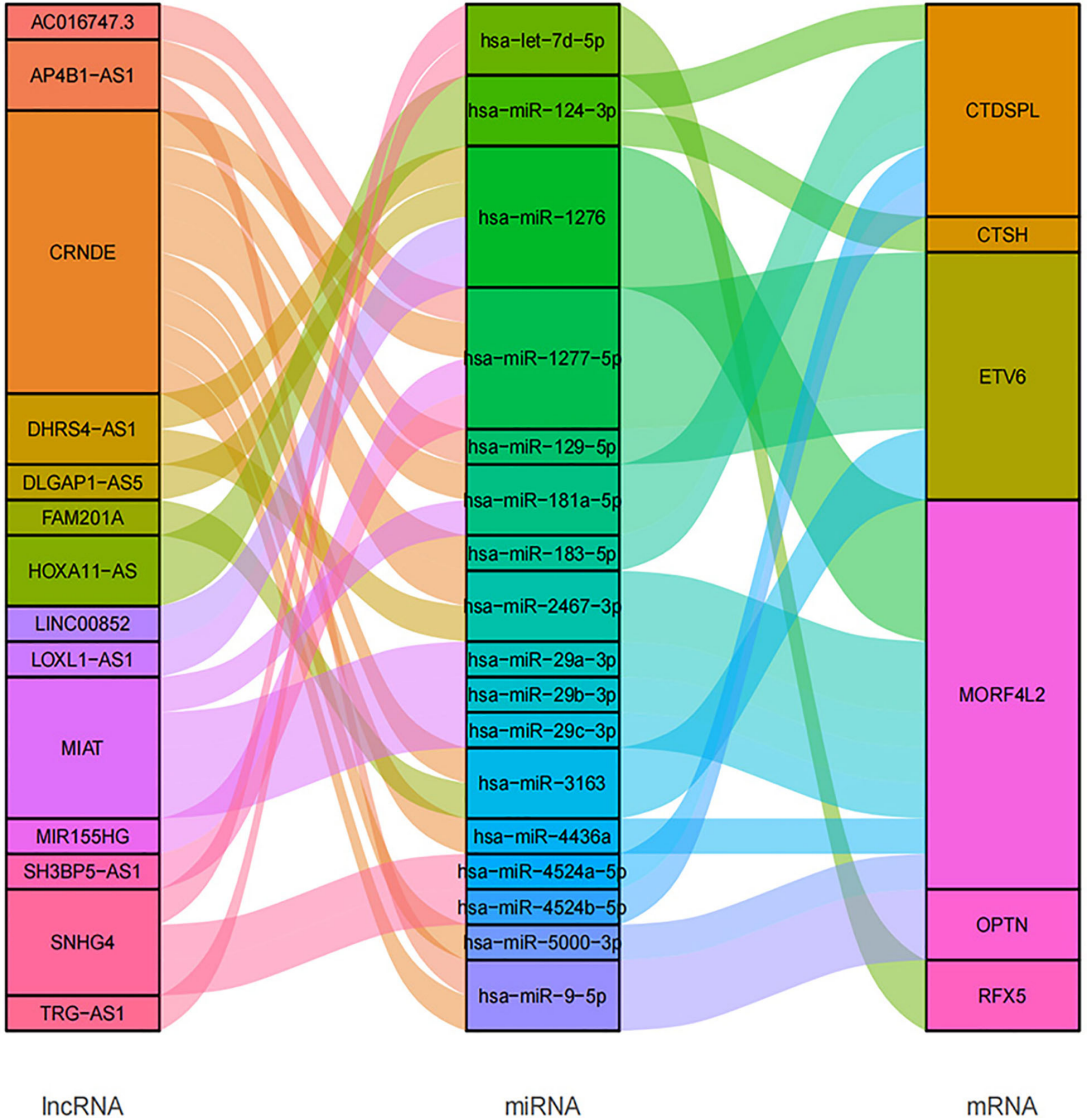
CeRNA network plot based on 11 prognosis signature genes and RT response related lncRNAs.

In total, we searched 52 miRNAs binding with 6 of 11 survival prognosis signature genes using **miRDB** and **mirTarbase** databases. Then, using another two RNA interaction databases **starBase** and **lncBase**, the 17 of 52 matched miRNAs had interaction with 14 targeted lncRNAs that were common in radio-sensitivity relevant lncRNA module (See **Figure 11**).

### Immune Cell Infiltration Analysis

**Figure 12:**
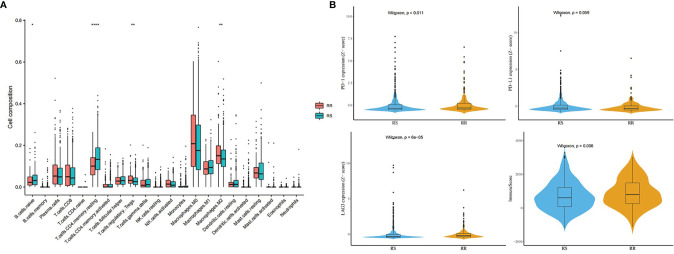
Immune analysis in the whole TCGA BRCA patients. **(A)** Distribution difference of immune cells between the RS group and the RR group. **(B)** Comparison of immune checkpoint genes and immune score between the RS group and the RR group (* means P-value <0.05; ** means P-value <0.01; **** means P-value <0.0001).

**Figure 12** shows the difference of TIICs between the RS group and the RR group in the whole TCGA BRCA patients, estimated by the TIMER2.0. The distributions of immune cells, namely, CD4^+^ T cells and B cells, enriched much in the RS group than in the RR group (**Figure 12A**). However, macrophages infiltrated much in the RR group. In several immune checkpoint related genes such as programmed death-1 (PD1) and Lymphocyte-activation gene 3 (LAG3), the RR group had higher expression level with higher immune score (**Figure 12B**).

## Discussion

Heterogeneity in terms of tumor characteristics, prognosis, and survival among cancer patients has been a persistent problem for many decades. A major issue in radiation therapy of cancer is predicting patient radio-sensitivity. Tumor molecular mapping has been used to develop radio-sensitive genetic signatures and has been used to identify prognostic or predictive biomarkers of radiation responses (7, 22, 23). In breast cancer patients, there is a strong correlation between tumor response and PFS. The treatment effect of tumor response well predicts the treatment effect of PFS, so PFS is an acceptable alternative endpoint of tumor treatment response (24).

In this study, we first used PFS as a feasible indicator of radiotherapy response in breast cancer patients with radiotherapy because of mass missing data for “treatment response” (missing rate: 80.15%). PFS is an alternative endpoint for OS to evaluate survival benefit. We selected patients who accepted a clear schedule of radiation therapy and considered those with a PFS event as RT non-response subjects. Patients with balanced clinical baseline information and longer non-progression survival time were selected as control subjects.

Then WGCNA algorithm was applied to explore the most relevant genes to the response between the two groups. Based on the module genes, we developed an effective survival prognosis gene signature of breast cancer patients to identify the benefit group of radiotherapy. This gene signature as an independent prognosis feature, could well predict the survival of patients with radiotherapy in both developed and validation datasets. Clinical decision based on the gene signature could offer more benefit for breast cancer patients. Importantly, our study found that prior patient stratification based on the gene signature could help clinicians to comprehensively decide which kind of patients are much preferable to receive radiotherapy and what patients should be spared toxicity-related to RT.

The acquired 11 signature genes were cadherin EGF LAG seven-pass G-type receptor 2 (CELSR2), creatine kinase B (CKB), cathepsin H (CTSH), ETS variant 6 (ETV6), CTD small phosphatase like (CTDSPL), monoacylglycerol acyltransferase 1 (MGAT1), mortality factor 4 like 2 (MORF4L2), optineurin (OPTN), regulatory factor X5 (RFX5), ST6 N-acetylgalactosaminide alpha-2,6-sialyltransferase 4 (ST6GALNAC4), and unc-93 homolog B1 (UNC93B1). When using online analysis tool STRING (https://www.string-db.org/) to perform protein to protein interaction, there were no interaction between these genes. Studies have reported that CELSR2, ETV6, MGAT1, and RFX5 were associated with breast cancer (25–28). CELSR2 is downregulated in breast cancers. ETV6–NTRK3 fusion gene is a type of genetic alterations associated with heterogeneity. MGAT1 takes part in aberrant N-glycan Golgi remodeling and metabolism which is associated with epithelial–mesenchymal transition (EMT). RFX5 can strongly increase transcriptional activity of LINC00504 and the latter is upregulated in breast cancer.

In this study, we tried to explore the gene regulatory CeRNA network of the 11 hub genes. CeRNA network shows the regulatory relationship of 14 relevant lncRNAs as ceRNA to 6 signature genes. These mRNAs and lncRNAs were associated with radiotherapy response. In the CeRNA network, we found that CRNDE participated in the binding of multiple miRNA regulatory axes. CRNDE (Colorectal neoplasia differentially expressed) is an oncogenic long non-coding RNA and has been demonstrated to be involved in multiple biological processes of different cancers, including breast cancer, which might be a potential diagnostic biomarker and prognostic predictor (29). Because of its participation in diverse oncogenic biological processes, CRNDE may illustrate the molecular heterogeneity of tumor. lncRNA MIAT (myocardial infarction associated transcript) originally has been considered as an lncRNA associated with a susceptibility to myocardial infarction. But later it was found to be related to cancers, involved in breast cancer progression (30).

Go functional enrichment analysis revealed that 534 MEbrown module genes were mainly enriched in immune-related processes. CTSH and UNC93B1 were enriched in “adaptive immune response”. Then we retrieved detailed biological annotation information of the term “adaptive immune response” using DAVID. The term is explained as “An immune response mediated by cells expressing specific receptors for antigen produced through a somatic diversification process, and allowing for an enhanced secondary response to subsequent exposures to the same antigen (immunological memory)”. Thus genes involved in this process may induce the change of immune micro-environment under the stimulus of the external environment (e.g., radiation).

Further immune estimated analysis showed the difference in the distribution of immune micro-environment between radio-sensitivity group and radio-resistant group. Specifically, CD4^+^ T cells and B cells, enriched much in the RS group than in the RR group. However, macrophages infiltrated much in the RR group. This phenomenon has been found in other studies (31). Infiltration of macrophages in solid tumors is associated with poor prognosis and correlates with chemotherapy resistance in most cancers (32). In addition, the RR group had higher immune score and expression level of PD1 and LAG3. PD-L1 on tumor cells may engage PD-1 receptors resulting in suppression of T-cell mediated immune response (33, 34). LAG3 is an inhibitory immune checkpoint of T cells that negatively regulates T cell proliferation, activation, and homeostasis (35). The varied component of micro-environment in the RR group may confer radiation resistance.

This study has its merits. We first used PFS as a feasible alternative indicator of radiotherapy response among breast cancer patients and accordingly established a survival prognosis gene signature which was proved to distinguish and predict radiotherapy benefit patients. The limitation of this study is that the sample size of the validation cohort (n = 130) was too small so that some results had not enough statistical power.

In conclusion, our study developed a strategy of building survival prognosis gene signature according to clinical treatment responsiveness PFS to identify and predict radiotherapy survival benefit in breast cancer patients. The 11-gene signature may reflect differences in the tumor immune micro-environment that underlie the differential response to radiation therapy and could guide clinical-decision making related to radiation in breast cancer patients. For precision medicine, our work offered more evidence and clues for using radiotherapy response related genes as potential signature to identify radio-sensitive for cancer patients or as targets that promote personalize radiation.

## Data Availability Statement

Publicly available datasets were analyzed in this study. This data can be found here: https://gdc.xenahubs.net https://www.ebi.ac.uk/arrayexpress/.

## Author Contributions

Study conception and design: JC, SL, and ZT. Data collection and clean: RG, XZ, and HL. Real data analysis and interpretation: JS, DY, and LB. Drafting of the manuscript: JS and DY. All authors contributed to the article and approved the submitted version.

## Funding

This work was supported in part by the National Natural Science Foundation of China (81773541), funded from the Priority Academic Program developed of Jiangsu Higher Education Institutions at Soochow University, the State Key Laboratory of Radiation Medicine and Protection (GZK1201919) to ZT, National Natural Science Foundation of China (U1967220 and 81872552) to JC, the Jiangsu higher education institution innovative research team for science and technology (2021), Key technology program of Suzhou people’s livelihood technology projects (Grant No. SKY2021029), Key programs of the Suzhou vocational health college (Grant No. szwzy202102), the Qing-Lan Project of Jiangsu Province in China (2021) to SL. The funding body did not play any roles in the design of the study and collection, analysis, and interpretation of data and in writing the manuscript.

## Conflict of Interest

The authors declare that the research was conducted in the absence of any commercial or financial relationships that could be construed as a potential conflict of interest.

## Publisher’s Note

All claims expressed in this article are solely those of the authors and do not necessarily represent those of their affiliated organizations, or those of the publisher, the editors and the reviewers. Any product that may be evaluated in this article, or claim that may be made by its manufacturer, is not guaranteed or endorsed by the publisher.

## Supplementary Material

Supplementary Figure 1 Process of searching the most relevant lncRNA module to RT response.

## Abbreviations

PFS, progression-free survival; OS, overall survival; WGCNA, weighted correlation network analysis; BRCA, breast invasive carcinoma; FPKM, Fragments per Kilobase per Million; RT, radiotherapy; PR, progesterone receptor; LASSO, Least Absolute Shrinkage and Selection Operator; MAD, median absolute deviation; TOM, topological overlap matrix; PCA, principal component analysis; RS, radio-sensitive; RR, radio-resistant; ROC, receiver operating characteristic; AUC, area under the curve; DCA, decision curve analysis; C-index, concordance index; GO, gene ontology; BP, biological process; MF, molecular function; CC, cellular component; CeRNA, Competing Endogenous RNAs; TIICs, tumor-infiltrating immune cells; DCs, dendritic cells; K–M, Kaplan–Meier; PD1, programmed death-1; PD-L1, programmed cell death 1 ligand 1.
