# Supplementary figures and images for: An 11-Gene Signature Based on Treatment Responsiveness Predicts Radiation Therapy Survival Benefit Among Breast Cancer Patients
Source: Front Oncol. 2022 Jan 6;11:816053. doi: 10.3389/fonc.2021.816053 (PMC8770413; doi:10.3389/fonc.2021.816053)

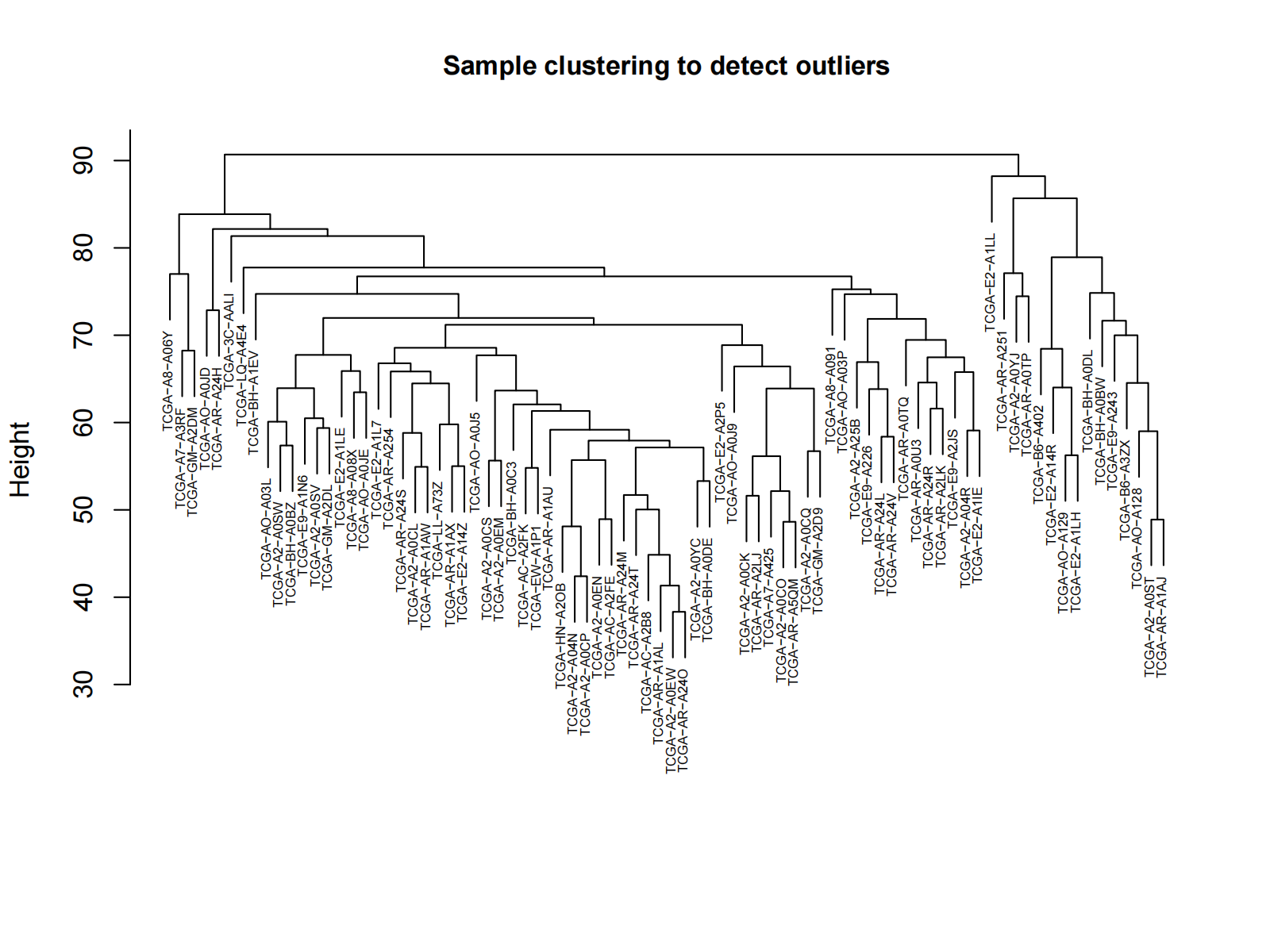


FigureS1A


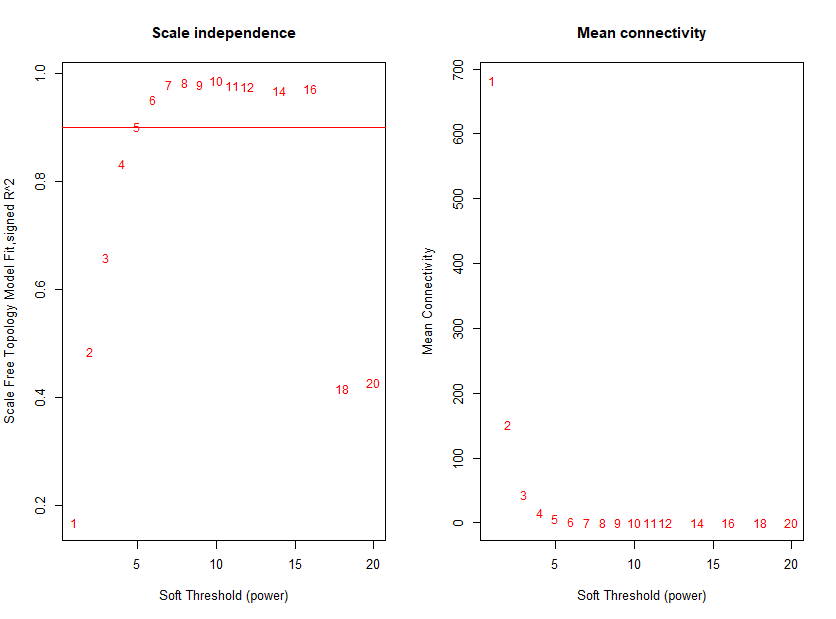


FigureS1B


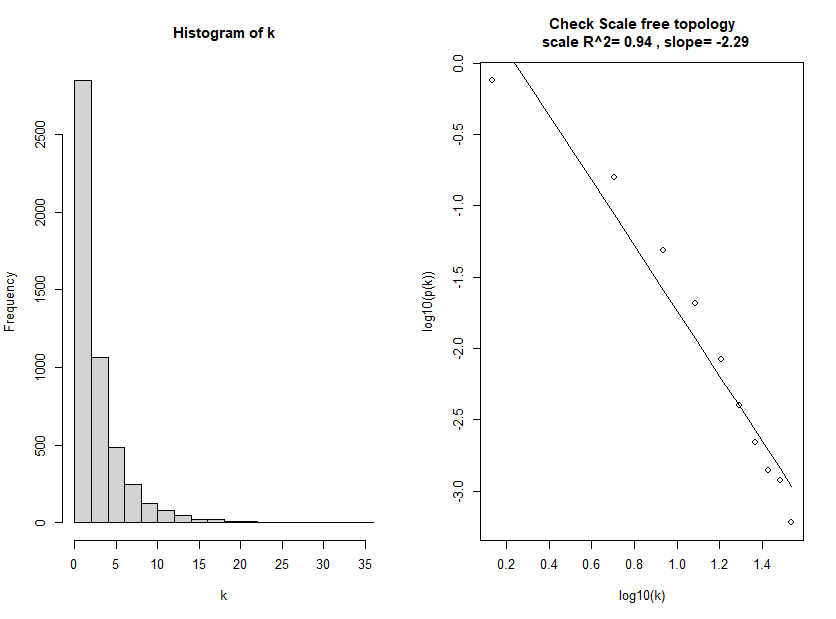


FigureS1C


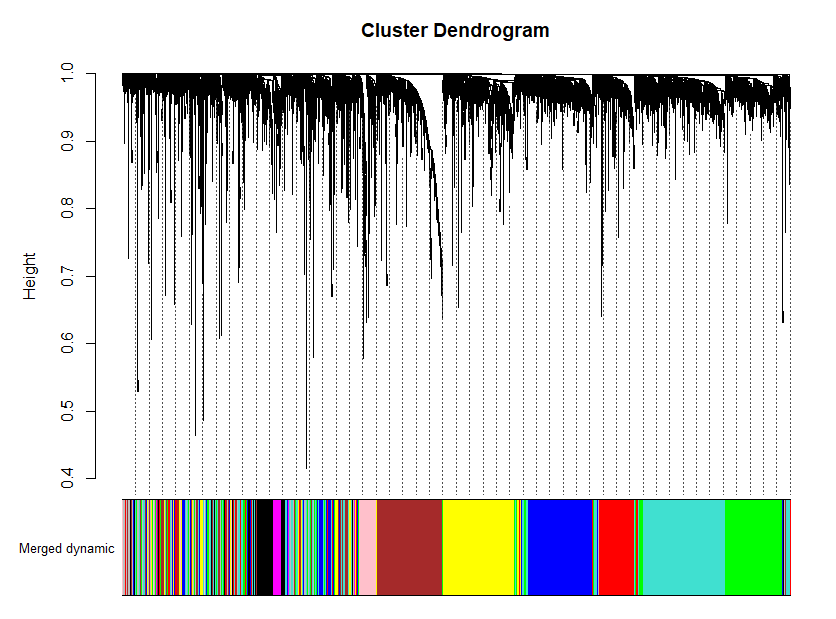


FigureS1D


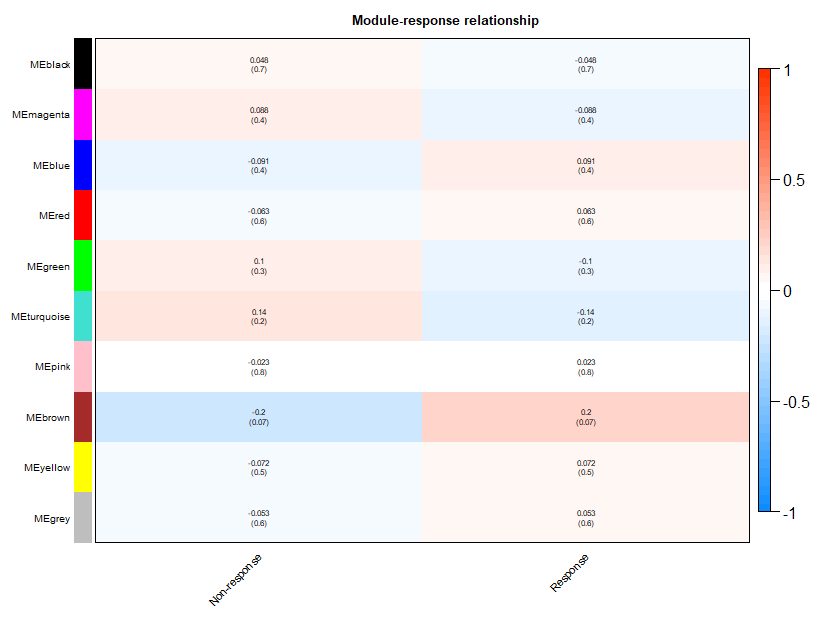


FigureS1E

Supplement: Supplementary Figure 1 — Process of searching the most relevant lncRNA module to RT response. [file DataSheet_1.docx]
