# Supplementary material for: An 11-Gene Signature Based on Treatment Responsiveness Predicts Radiation Therapy Survival Benefit Among Breast Cancer Patients
Source: Front Oncol. 2022 Jan 6;11:816053. doi: 10.3389/fonc.2021.816053 (PMC8770413; doi:10.3389/fonc.2021.816053)
Supplement: Supplementary file 2 [file Table_1.docx]

**TableS1.** Clinical factors in RT response group and RT non-response group.

| Characteristic |  | Responder  (N=41) | Non-responder  (N=41) | *P*^*^ |
| --- | --- | --- | --- | --- |
| Chemotherapy | yes | 39 (95%) | 40 (98%) | >0.9 |
|  | no | 2 (5%) | 1(2%) |  |
| Age | <60 | 23 (56%) | 19 (46%) | 0.4 |
|  | >=60 | 18 (44%) | 22 (54%) |  |
| PR status | positive | 30 (73%) | 25 (66%) | 0.5 |
|  | negative | 11 (27%) | 13 (34%) |  |
|  | unknown | 0 | 3 |  |
| N Stage | N0/N1/N2 | 30 (73%) | 23 (57%) | 0.14 |
|  | N3 | 11 (27%) | 17 (42%) |  |
|  | unknown | 0 | 1 |  |
| Pathological stage | I/II | 25 (61%) | 19 (46%) | 0.2 |
|  | III/IV | 16 (39%) | 22 (54%) |  |

^*^Pearson's Chi-squared test; Fisher's exact test .
